# Ginger (*Zingiber officinale*) and Zingerone Antioxidant Properties Studied Using Hydrodynamic Voltammetry, Zingerone Crystal Structure and Density Functional Theory (DFT)—Results Support Zingerone Experimental Catalytic Behavior Similar to Superoxide Dismutases (SODs)

**DOI:** 10.3390/ijms262110645

**Published:** 2025-10-31

**Authors:** Miriam Rossi, Taylor S. Teitsworth, Elle McKenzie, Alessio Caruso, Natalie Thieke, Francesco Caruso

**Affiliations:** 1 Chemistry Department, Vassar College, Poughkeepsie, NY 12604, USA; 2 Department of Chemistry and Chemical Biology, Harvard University, Cambridge, MA 02138, USA

**Keywords:** zingerone, superoxide scavenging, RRDE voltammetry, DFT mechanism, antioxidant, SOD mimic

## Abstract

Ginger is a common spice found in many cuisines all over the world that is from the rhizome of *Zingiber officinale*. Additionally, it has been used in traditional medicinal practices as an aid in many ailments ranging from nausea to muscle pain. The non-volatile compounds of ginger, including zingerone, are responsible for pungency and they have widespread biomedical activities. The crystal structure of zingerone, a 6-gingerol degradation product and phenolic compound, reveals that the C4 hydroxyl group is the fulcrum for strong intermolecular interactions such as (O1-H2…O3) 2.737(2) Å. Our electrochemical results using rotating ring-disk electrode (RRDE) hydrodynamic voltammetry demonstrate that zingerone is an effective scavenger of superoxide radical anions and that zingerone, unlike powdered ginger, is a strong antioxidant with a collection efficiency slope of −6.5 × 10^4^ M^−1^. The addition of vitamin C enhances scavenging activity for both zingerone and ginger powder, although the effect is more noticeable with zingerone. Correspondingly, the zingerone/vitamin C efficiency slope value is −5.40 × 10^5^ M^−1^. Density Functional Theory (DFT) calculations permit the development of a plausible antioxidant mechanism for zingerone, and zingerone synergistic action with vitamin C, in which zingerone is capable of being regenerated with the assistance of protons that may be provided by ascorbic acid. This mechanism demonstrates that zingerone acts as a strong antioxidant agent by virtue of its C4 hydroxyl group and aromatic system. The scavenging chemical reaction is the same as that obtained through the dismutation of superoxide by superoxide dismutase (SOD) enzymes into hydrogen peroxide and molecular oxygen. Thus, zingerone behaves as a SOD mimic.

## 1. Introduction

Ginger (*Zingiber officinale* Roscoe), which, along with turmeric (*Curcuma longa*) and cardamom (*Elettaria cardamomum*), belongs to the Zingiberaceae plant family, is commonly used in cuisines all over the world. Its origins can be traced back over 5000 years ago to India and China [1,2,3,4], where it is commonly used in Ayurvedic and traditional Chinese medicines. Ginger is grown in tropical and subtropical climates [5]. Today, ginger products are used as herbal remedies to treat a wide set of ailments ranging from muscular aches and pains to nausea [6]. Interestingly, dried ginger powder appears to have increased biological activity over fresh [2,3,7]. Current scientific studies on ginger have established that it has a wealth of beneficial properties including anti-inflammatory, antiemetic, antimicrobial, antioxidant, anticancer and anticoagulant [8]. The role of ginger and its phenolic compounds in the immune system is covered in several thorough review articles [9,10].

**Figure 1:**
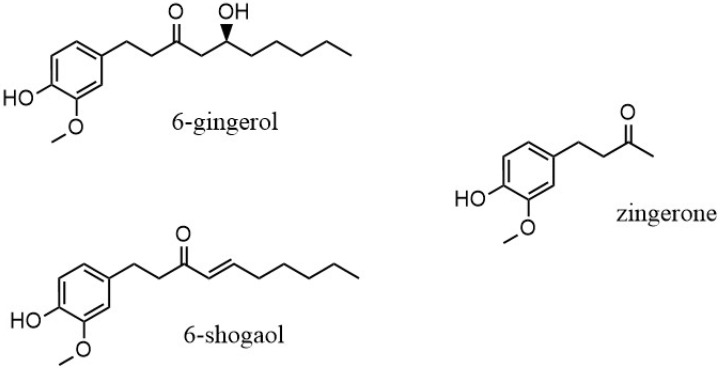
6-gingerol and degradation products, 6-shogaol and zingerone.

The rhizome is the part of the *Zingiber officinale* plant that is used, both fresh and as dry powder. The chemical composition of the ginger rhizome has been thoroughly studied, with more than 60 active constituents identified. Compounds found in ginger are organized into two main categories: volatile and non-volatile [2]. The non-volatile compounds of ginger include gingerols, shogaols, paradols and zingerone, and they are the representative bioactive constituents of ginger, making up 1–6% of fresh ginger by weight. The pungent flavors and odors of these non-volatile compounds give the distinctive scent and taste to ginger [7]. The gingerols are differentiated by the length of their unbranched alkyl chains, present as 6-, 8- and 10-gingerol. Of these, 6-gingerol is the most abundant (about 6% *w*/*w*), although it is thermally unstable because of its β-hydroxy keto group [6]. When heat is applied, gingerol degrades to the corresponding shogaol through a dehydration process. Through a retro aldol reaction, the alkyl chain is cleaved and results in the formation of zingerone, (4-(4-hydroxy-3-methoxyphenyl)butan-2-one) and C_11_H_14_O_3_ (Figure 1) [11]. As a result, zingerone concentration in dried ginger is present in a greater amount. Recent gas chromatography/mass spectroscopy analysis of ginger demonstrated that zingerone was one of the pungent compounds [12].

Zingerone is interesting to study because it is readily available and has low acute toxicity, with oral LD_50_ values of 2580 mg/kg (rat) [13]. Zingerone has been used therapeutically including as a chemopreventive agent in model experimental colon carcinogenesis in vivo studies on Wistar rats [14], in the treatment of liver diseases [15,16] and as a neuroprotective agent [17]. Recent reviews on zingerone pharmacological activities list a plethora of biomedical conditions that are alleviated through the use of zingerone [11,18].

Numerous reports of zingerone’s pharmaceutical properties discuss its therapeutic function in line with its antioxidant and anti-inflammatory capacities. These include studies that determine its usefulness as a food antioxidant ingredient based on decreased peroxidation of phospholipid liposomes in the presence of iron(III) and ascorbate [19]. Also, zingerone demonstrated protective antioxidant effects against DNA damage in an in vitro study [20]. Likewise, both in vivo and in vitro studies on septic mice demonstrate that zingerone administration diminishes reactive oxygen species (ROS) concentration and results in systematic inflammation [21]. Zingerone reduction of oxidative stress and inflammation is also valuable in damage to kidney tissue caused by arsenic poisoning [22]. Because insight into the antioxidant mechanism of the biologically active compounds in ginger is limited [12], in this study, we aimed to clarify the chemical mechanism by which zingerone, a less studied ginger compound, can act as a scavenger of ROS.

Ginger antioxidant activity using DPPH methods is reported in the literature [23] and interestingly, dried ginger showed higher antioxidant activity [24]. Although widely used for their simplicity, DPPH assays are problematic since DPPH is a stable, large lipophilic radical that is not found in biological systems [25] and is therefore not a good model system for biomedical ROS scavenging studies. Also, to assess scavenging activity by antioxidants, the concentration of the superoxide anion (whose disappearance we are trying to measure) is needed. A significant advantage with using rotating ring-disk electrode voltammetry (RRDE) is that O_2_^•−^ is made in situ and the RRDE method measures superoxide scavenging directly, e.g., a real superoxide concentration is detected.

Scavenging of excess ROS is an important role that is carried out very effectively by endogenous enzymes such as superoxide dismutases (SODs). These are organometallic enzymes and their mechanism involves the dismutation of O_2_^•−^ to H_2_O_2_ and O_2_ at their active site through the action of redox-active metal ions [26]. Additionally, these enzymes are assisted in their ROS scavenging by ingested natural food products that contain polyphenolic compounds. Our laboratory has reported on the antioxidant activity of several natural compounds and nutritional foods [27,28,29]. We have determined that some plant-based polyphenolic antioxidants act as SOD mimics through suitable intermolecular interactions such as π-π stacking and hydrogen bonding [30]. Notably, the isoflavone formononetin [29] and kaempferol [28] can release the O_2_ molecule, regenerate themselves and prepare for another cycle, thus acting as SOD mimics.

Since no prior work has measured zingerone antioxidant activity or elucidated zingerone’s antioxidant mechanism, this work aims to determine the mechanism of superoxide scavenging by zingerone through combined electrochemical and DFT analyses.

We experimentally measure the scavenging ability of zingerone and powdered ginger towards the superoxide anion free radical, a common and reactive ROS in biological systems using RRDE. We then obtain a plausible mechanism for superoxide scavenging, which correlates with experimental results, through Density Functional Theory (DFT) analyses. From the zingerone crystal structure, we obtain a relationship between its molecular structure, its intermolecular interactions and its antioxidant activity. Additionally, we evaluate possible synergistic action between zingerone and vitamin C and explain our experimental results with the DFT-derived mechanism.

## 2. Results and Discussion

### 2.1. X-Ray

Our zingerone X-ray data were collected at low temperature (125 K) and therefore of high quality. Using low temperature during data collection reduces atomic thermal motion and yields a sharper view of the electron density and improved identification of hydrogen atoms, and a clearer definition of atoms participating in intermolecular interactions [31].

**Figure 2:**
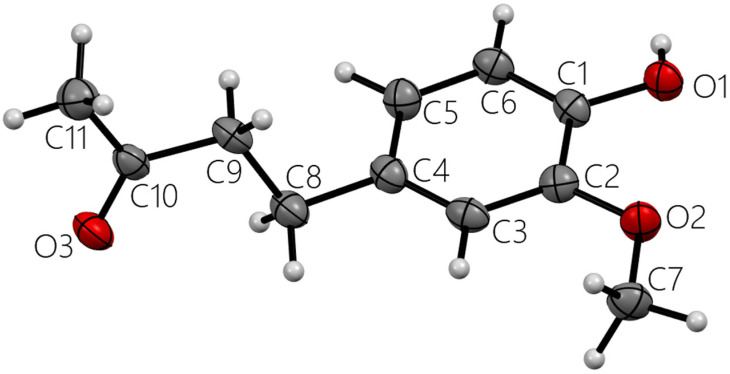
Single molecule zingerone. All heavy atoms labeled.

**Figure 3:**
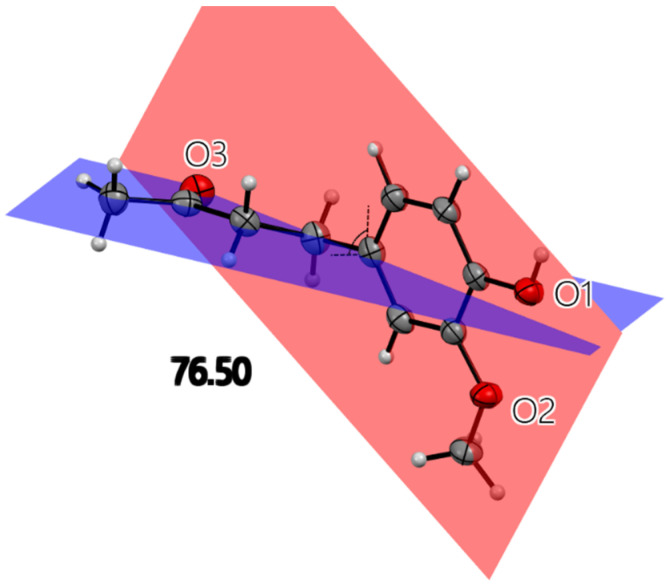
Single molecule of zingerone with three oxygen atoms labeled. All heavy atoms lie on one of the two planes illustrated. The angle between planes is 76.5°. The red plane contains aromatic ring, hydroxyl and methoxy oxygen atoms. The blue plane contains an aliphatic chain.

**Figure 4:**
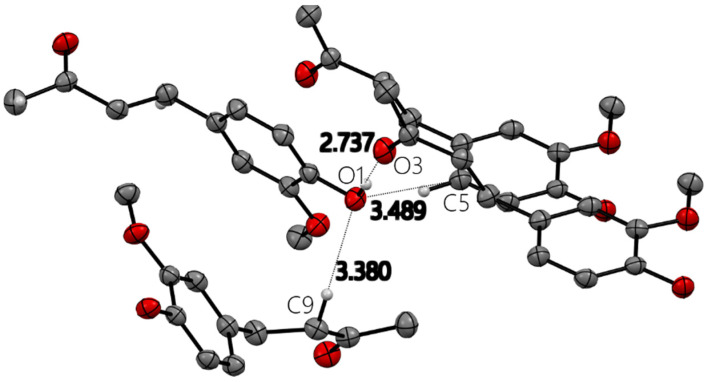
Four zingerone molecules with hydrogen bond and close contact distances (Å) around hydroxyl oxygen O1 in zingerone X-ray molecular structure. Only relevant H atoms are shown for clarity.

All X-ray molecular drawings were created with Mercury 2022.3.0 from the Cambridge Crystallographic Data Centre (CCDC) [32]. The zingerone molecule itself (Figure 2) can be described as having two planes, namely, one containing the aromatic ring and the other for the aliphatic tail, as seen in Figure 3. All non-hydrogen atoms lie on one of the two planes, with the angle between the two being 76.5°. The molecules are connected by a very strong hydrogen bond between the phenyl hydroxyl group and the carbonyl oxygen atom at an O…O distance of (O1-H2…O3) 2.737(2) Å. This is shown in Figure S1, Supplementary Materials. This hydrogen bond connects the molecules in a twisted infinite ribbon. Additionally, the hydroxyl oxygen is responsible for two other longer hydrogen bond interactions, as a hydrogen bond acceptor, with two other molecules to form a three-dimensional array, as shown in Figure 4. All distances and angles of the intermolecular interactions are included in Table 1.

The zingerone structure had been resolved earlier at room temperature and is available in the CCDC with the code FOZXUQ [33]. Additionally, the zingerone molecule bears some resemblance to another compound found in Zingiber species, tetrahydrocurcumin (zingerone being about half of a tetrahydrocurcumin molecule). The crystal structure of tetrahydrocurcumin is in the CCDC [32] with the code EYIZIV [34] and it shares some similarities with zingerone in that the aliphatic portion of the molecule is at a large angle (89°) from the aromatic portion. In addition, the hydroxyl group in tetrahydrocurcumin is responsible for the two hydrogen bonds, including one with the carbonyl group of an adjacent molecule as seen in zingerone. Other natural products having the same 4-hydroxy and 3-methoxy substitution pattern on the phenyl ring are vanillin (CCDC code: YUHTEA07) [35] and ferulic acid (CCDC code: GASVOL01) [36]. In both cases, the phenolic hydroxyl group is involved in one hydrogen bond and two short contacts with adjacent molecules.

The strong hydrogen bonding and close contacts shown by the C1 hydroxyl group in the zingerone crystal structure (Figure 4), and described in Table 1, reveal the tendency of the hydroxyl group to interact with the electron-rich moieties. These structural features give insight into how zingerone can behave as a scavenging agent towards the superoxide radical anion and act as an antioxidant.

### 2.2. RRDE

#### 2.2.1. Zingerone

**Figure 5:**
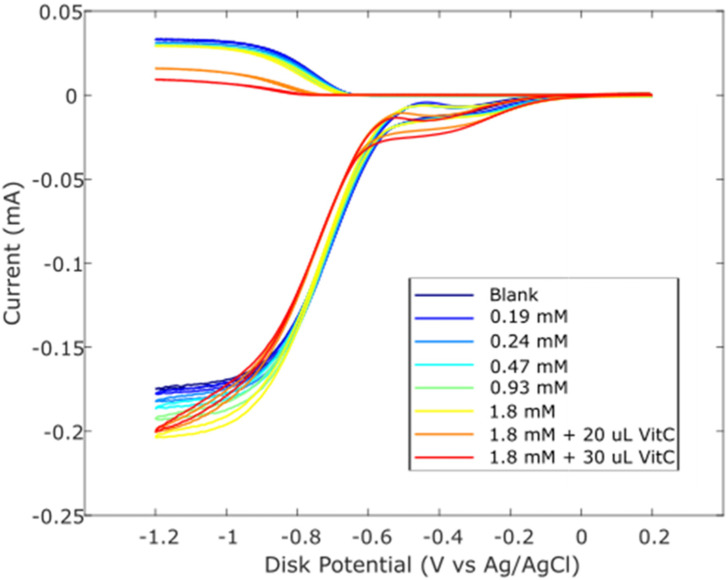
RRDE voltammograms after additions of 0.03 M zingerone and 0.03 M vitamin C. Blank plus first five aliquots are the concentrations of zingerone used. To the 1.8 mM zingerone solution, 20 μL of 0.03 M vitamin C was added (0.012 mM) (orange); the last experiment (red) has 50 μL total vitamin C added (0.03 mM).

**Figure 6:**
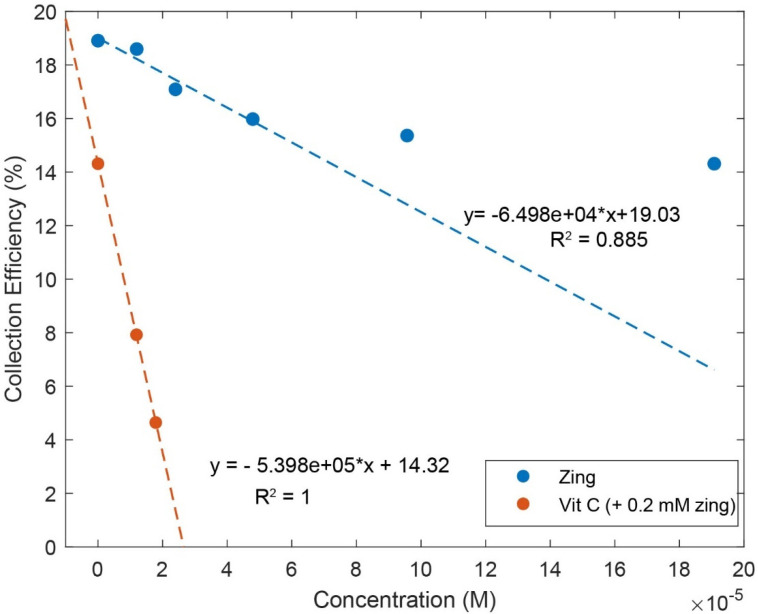
Collection efficiencies for solutions containing zingerone (“zing”) only (blue line) and solutions containing both zingerone and vitamin C (red line). For comparison, the slope of pure vitamin C is −2.6 × 10^4^ M^−1^ [27]. The steeper slope for the red line shows that the greater the rate of change, the more effective the antioxidant activity. For zingerone line expression, only the first four points were included.

Zingerone was completely dissolved in DMSO 0.03 M, and aliquots of this solution were added to the electrolytic cell as described in Hydrodynamic Voltammetry (RRDE). The resulting voltammograms are shown together in Figure 5, and the calculated collection efficiency is presented in Figure 6. We see that the zingerone collection efficiency slope (−6.5 × 10^4^ M^−1^), a measure of antioxidant activity, is similar to that of several isoflavones [29] and weaker than that of butein (slope = −11.2 × 10^4^ M^−1^) [37].

Earlier studies in our laboratory demonstrated that the acidic proton of ascorbic acid reinforced the antioxidant capability of vitamin E [27] and kaempferol [28]. So, we added vitamin C to our zingerone solution and observed that the addition of vitamin C greatly enhanced the antioxidant action. The effect is readily visible in the RRDE voltammograms and the change in collection efficiency slope is quite dramatic, −5.4 × 10^5^ M^−1^. Vitamin C alone has a slope of −2.6 × 10^4^ M^−1^ [27] and comparing it to that of zingerone (red line), which has a slope of −6.5 × 10^4^ M^−1^, as shown in Figure 6, the latter is a better scavenger. However, the combination of vitamin C and zingerone produces a much stronger scavenging outcome (Figure 6; red line with slope −5.4 × 10^5^ M^−1^) and a marked synergy effect. We report the greater than sevenfold increase in slope, [(−5.4 × 10^5^) − (−6.5 × 10^4^)]/(−6.5 × 10^4^) = 7.3, and note that it is greater than the sum of the two individual slopes for vitamin C and zingerone (i.e., −5.4 × 10^5^ > −6.5 × 10^4^ + (−2.6 × 10^4^)). A DFT analysis, in Section 2.3, will describe a potential molecular mechanism.

Observation of zingerone collection efficiency data in Figure 6 shows that the initial linear behavior becomes asymptotic. This behavior was observed previously for some antioxidants, for instance, quercetin and clovamide [38], whereas other antioxidants are linear for all ranges used in the method, e.g., isoflavones [29]. The explanation for this nonlinear behavior is an interaction between an antioxidant/superoxide intermediate and superoxide [38]. Since zingerone shows this nonlinear behavior, only the first four points were included to calculate the slope (Figure 6).

#### 2.2.2. Ginger Powder

**Figure 7:**
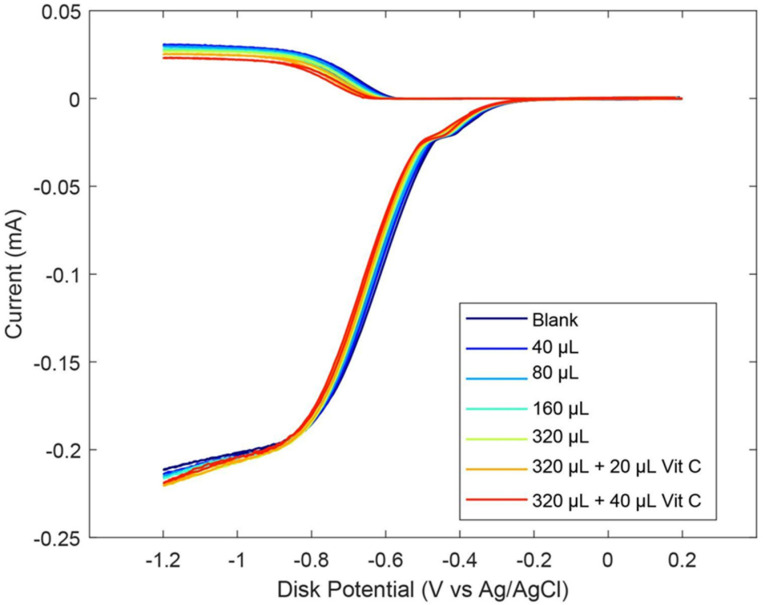
RRDE voltammograms after additions of 20.7 mg/mL ginger powder and 0.03 M vitamin C. Blank plus first five aliquots are volume of ginger used. To the 320 μL ginger solution, 20 μL of 0.03 M vitamin C was added (0.012 mM) (orange); the last experiment (red) has 50 μL total vitamin C added (0.03 mM).

**Figure 8:**
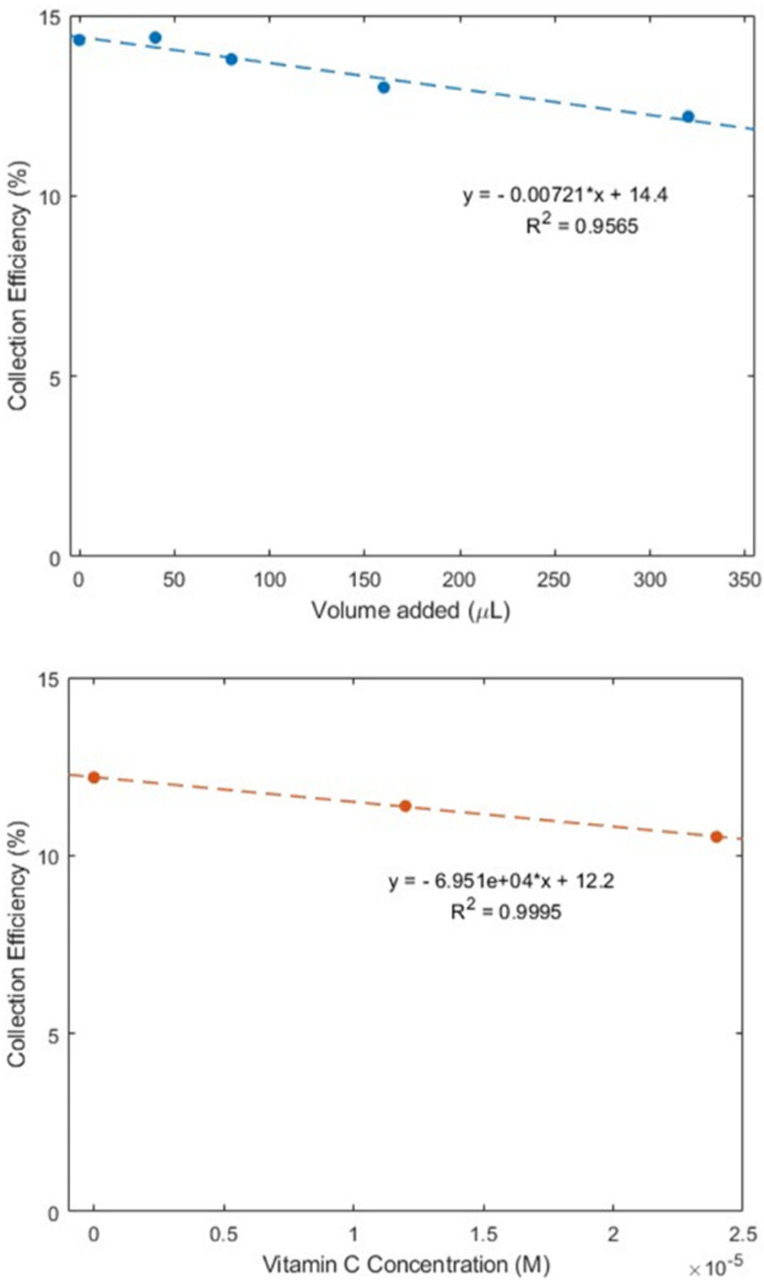
Collection efficiencies for (**top**) solutions of varying ginger volume added and (**bottom**) 320 μL of ginger (0.13 mg/mL) solution with added vitamin C.

As described earlier in the Introduction section, ginger powder contains a higher concentration of zingerone than fresh ginger. Our next experiment was conducted using ginger powder, 20.7 mg/mL DMSO. The extracted supernatant of the ginger–DMSO solution was analyzed using the RRDE method (Figure 7), and the collection efficiency for the aliquots is shown in Figure 8. We see that ginger powder demonstrates a lower capacity for scavenging the superoxide anion than zingerone. The addition of vitamin C increases ginger powder scavenging capacity, as seen in Figure 7 and Figure 8. The consequence of adding vitamin C to ginger, a natural product, is seen by comparing the efficiency for ginger added to vitamin C (Figure 8 right; slope = −6.9 × 10^4^ M^−1^) with vitamin C alone (−2.6 × 10^4^ M^−1^) [27]. This effect is also shown in Figure S2. On the other hand, ginger alone—slope −7.2 × 10^−3^ (Figure 8 left)—is weaker than the scavenging capacity of olive oil (−8.3 × 10^−2^ [39]).

Overall, our experimental results show that zingerone is a stronger antioxidant than ginger powder and that there is an apparent synergistic effect between the zingerone and vitamin C. In fact, our DFT-derived mechanism reveals that ascorbic acid provides the necessary protons for zingerone scavenging action, as shown in Section 2.3.

An in vivo experiment showing improvement of rat immune function and blood parameters upon administration of zingerone and vitamin C demonstrates synergistic action between the two compounds [40]. Also, in vivo trials on rats undergoing radiation treatments demonstrate less damage when ginger and vitamin C are given [41].

### 2.3. DFT

**Figure 9:**
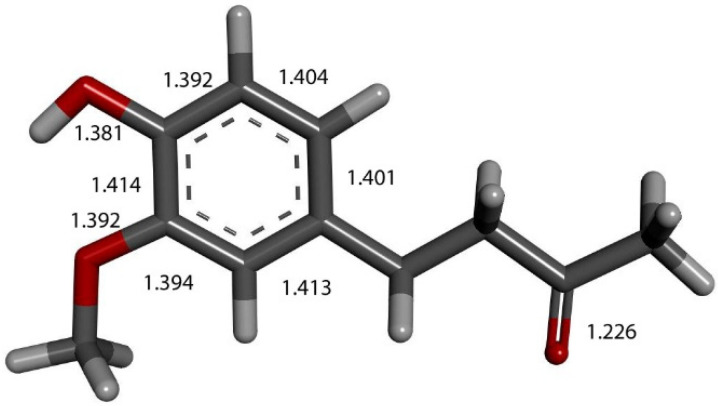
X-ray crystal structure coordinates of zingerone were input in Dmol^3^ and geometrically optimized. Indicated bond lengths were later compared with zingerone derivatives. Separation between H(hydroxy) and O(methoxy) is 2.121 Å, not indicated in figure for clarity.

**Figure 10:**
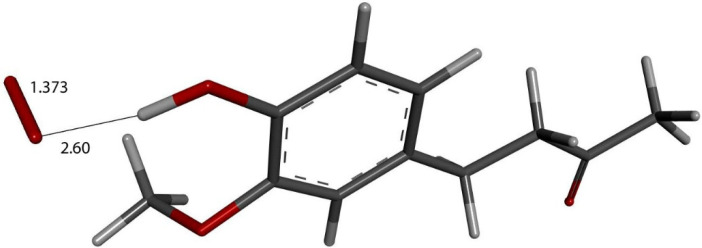
Zingerone van der Waals σ interaction with superoxide (red stick), 2.60 Å, initial state for DFT optimization.

**Figure 11:**
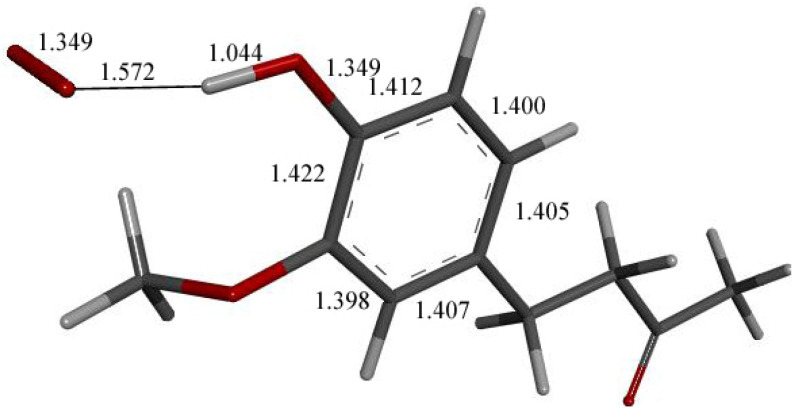
DFT optimization of Figure 10 arrangement.

**Figure 12:**
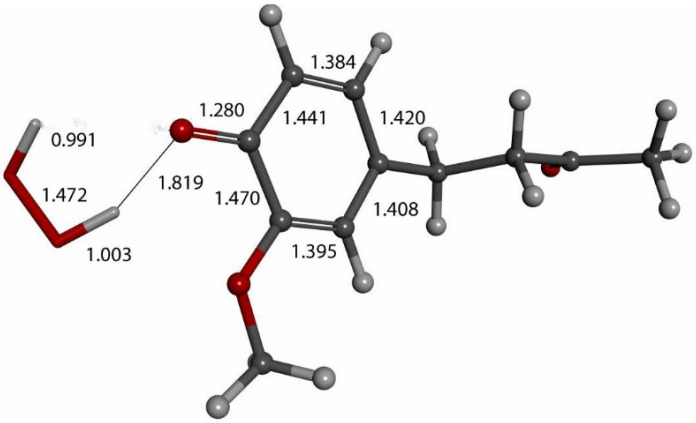
Result of DFT optimization after placing a proton near HO_2_^−^ anion in Figure 11 arrangement, which shows H_2_O_2_ formation (in stick mode) and H-bond to zingerone semiquinone (ball and stick mode), 1.819 Å. In fact, C4-O4 bond, 1.280 Å, is much shorter than 1.394 Å in Figure 9.

**Figure 13:**
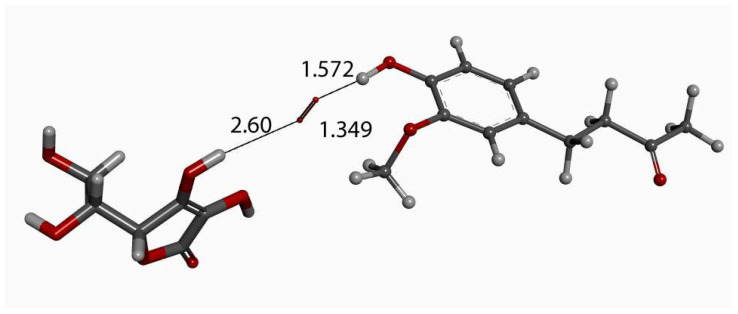
Exploration of possible vitamin C (wide stick) proton release through superoxide moiety (narrow stick) approach of zingerone (ball and stick) arrangement, as in Figure 3, in order to obtain a similar outcome as that in Figure 12. Initial state is shown here with van der Waals separation of 2.60 Å between ascorbic acid and superoxide.

**Figure 14:**
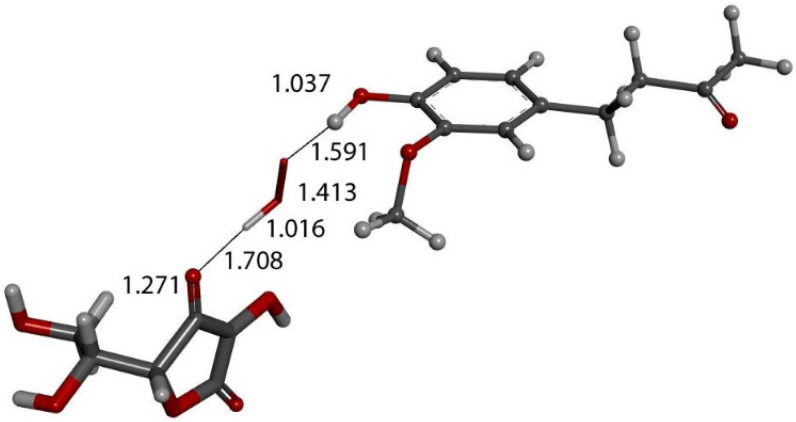
DFT optimization of Figure 13 arrangement shows HO_2_**^−^** anion formed, separated from zingerone, 1.591 Å, and ascorbate, 1.708 Å, showing that zingerone is insensitive to ascorbic acid attack.

We input the X-ray coordinates of zingerone in our Dmol^3^ system to obtain the resulting minimum of energy structure (Figure 9), and it is very similar to that in the crystal. The main difference is due to the different environment of the hydroxy group, which includes the neighboring keto group from another molecule in the crystal through the H-bond; see Section 2.1. This does not exist in the isolated molecule studied with DFT, and the hydroxyl group establishes an intramolecular H-bond to the methoxy group—D(H-O) = 2.121 Å. Our first approach to studying the interaction between the superoxide radical anion and zingerone began with the van der Waals separation between superoxide and the hydroxyl group of zingerone (Figure 10). The resulting product of DFT optimization shows a H-bond between both groups, as seen in Figure 11, and so, at this stage, there is no extraction of the proton by superoxide. Our next calculation explored the action of an acidic proton, resulting in the formation of a molecule of H_2_O_2_, separated by 1.819 Å (Figure 12). An equivalent study was performed using ascorbic acid to replace the isolated proton, with the initial arrangement shown in Figure 13. DFT optimization of this structural complex resulted in the superoxide radical capturing the acidic proton, along with the formation of ascorbate separated by 1.708 Å from the HO_2_ group, but the hydroxyl group of zingerone was not abstracted (Figure 14).

**Figure 15:**
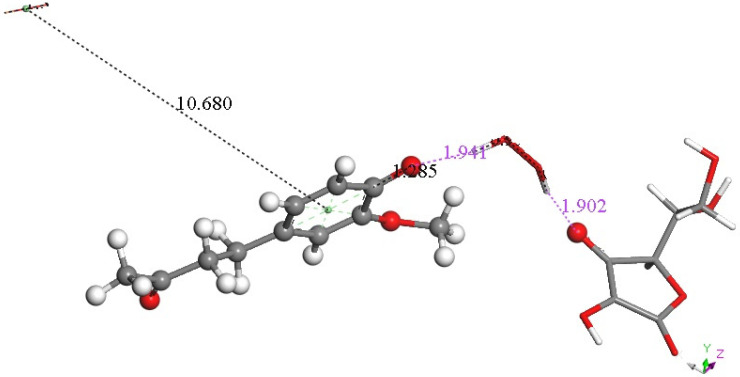
Result of DFT geometry optimization where the initial state has a superoxide π-π approaching the aromatic ring with 3.50 Å between centroids of the Figure 14 arrangement. A molecule of O_2_ (stick, upper left) is released, 10.680 Å, while H_2_O_2_ (stick) forms, well separated from zingerone semiquinone (ball and stick), 1.941 Å (violet), and ascorbate, 1.902 Å (violet). Also, shortening of ascorbic acid C4-O4 is seen, 1.285 Å. Since two superoxide radicals and a proton from ascorbic acid are present, this arrangement is that of a non-radical anion.

In previous studies [28,29] we observed that superoxide can approach scavengers using a π-π interaction and so we explored this possibility using the arrangement shown in Figure 14 with a second superoxide π-π stacked on zingerone, with separation between aromatic ring and superoxide centroids of 3.50 Ả. The resulting DFT optimization (Figure 15) shows a dramatic change: the zingerone hydroxyl releases its proton to form H_2_O_2_, which is well separated from ascorbate (1.941 Å) and zingerone anion (1.902 Å), whereas the second reacting π-π superoxide is rejected, showing separation between centroids of 10.680 Å. We concluded that the electron from the second superoxide was given to the zingerone ring, and this induced H_2_O_2_ formation plus O_2_.

**Figure 16:**
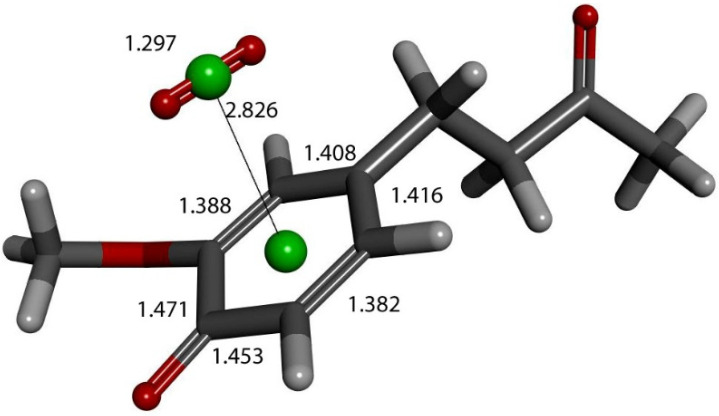
Product of further exploration of proton action in Figure 12 arrangement. After elimination of H_2_O_2_, zingerone semiquinone has superoxide π-π van der Waals placed above the ring, with separation between their centroids (green) of 3.50 Å. This is markedly shortened upon DFT optimization, 2.826 Å. Comparison with Figure 9 shows elongated bond lengths for C3-C4 (1.471 Å) and C4-C5 (1.453 Å), and shortening of C2-C3 (1.388 Å) and C5-C6 (1.382 Å) bond lengths.

Further exploration of the proton action in the previous Figure 12 arrangement was also made after elimination of H_2_O_2_, and zingerone semiquinone has superoxide π-π van der Waals placed above the ring, with separation between their centroids of 3.50 Å. This is markedly shortened upon DFT optimization, 2.826 Å (Figure 16). Comparison with Figure 9 shows elongation of bond lengths C3-C4 (1.471 Å) and C4-C5 (1.453 Å), compared to 1.414 Å and 1.392 Å, respectively. In addition, the C2-C3 (1.388 Å) and C5-C6 (1.382 Å) bond lengths become shorter than in Figure 9, 1.394 Å and 1.404 Å, respectively. This is consistent with a *para*-quinone-like arrangement for a conjugated single/double C-C bond configuration.

**Figure 17:**
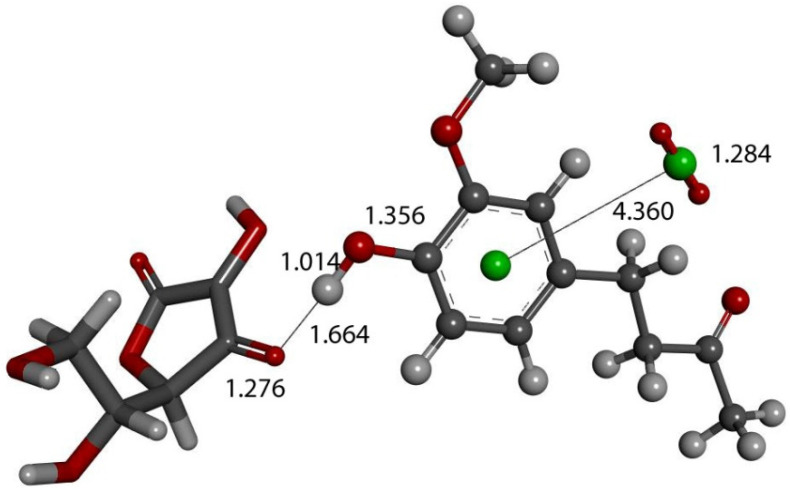
Vitamin C (stick) is placed at van der Waals separation, 2.60 Å, from O4 of the η-O_2_-zingerone semiquinone (ball and stick) shown in Figure 16. DFT geometry optimization shows a molecule of O_2_ (O-O distance = 1.284 Å) (ball and stick, on right) leaving the aromatic ring (green, centroid–centroid separation of 4.360 Å, greater than initial van der Waals distance, 3.50 Å) and zingerone being restored. This is seen in Supplementary Materials, Video S1.

The action of a second proton on the zingerone derivative anion (Figure 16) shows reformation of zingerone. Moreover, by replacing the proton with ascorbic acid (a weak acid) in the last DFT optimization, vitamin C releases its proton, generating ascorbate anion plus reformed zingerone (Figure 17). Input of this DFT optimization was performed by placing vitamin C at van der Waals separation, 2.60 Å, from O4 of the η-O_2_-zingerone complex shown in Figure 16. The DFT results show a molecule of O_2_ (O-O distance = 1.284 Å) leaving the aromatic ring (centroid–centroid separation of 4.360 Å, greater than the initial van der Waals distance, 3.50 Å) and zingerone being restored (Figure 17). A related video showing this reaction can be seen in Video S3. The whole process can be summarized by Equation (1), and so, zingerone behaves as a SOD mimic.

zingerone + 2 superoxide + 2 Vit C **→** [reformed zingerone] + H_2_O_2_ + O_2_ + 2 ascorbate (1)

The only difference with using a stronger acid than vitamin C is seen by comparing Figure 12 and Figure 14: the proton (of the stronger acid) forms H_2_O_2_ directly (Figure 12), whereas ascorbic acid is unable to do so (Figure 14). However, in this case, the arrival of a second superoxide (π-π approached) induces H_2_O_2_ formation, as seen in Figure 15.

**Figure 18:**
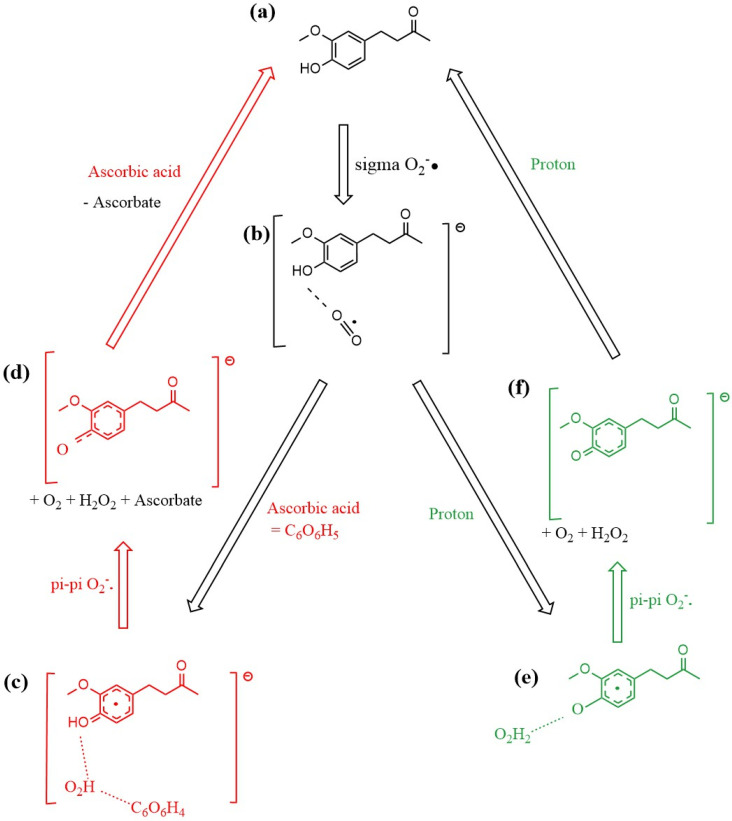
Mechanism of zingerone antioxidant action for scavenging the superoxide radical. (**a**) zingerone; (**b**) σ interaction between superoxide and zingerone hydroxyl in position 4 establishes a H-bond (1.591 Å in Figure 11); (**c**) red color: ascorbic acid releases its proton after interaction with the most exposed O atom of superoxide, forming the HO_2_ species which interacts through H-bond with H(4) of zingerone (1.591 Å) and ascorbate (1.708 Å), as seen in Figure 14, that is, zingerone still holds its 4-OH proton insensitive to this attack; (**d**) upon π-π interaction of a second superoxide, H_2_O_2_ forms, well separated from zingerone O(4), 1.941 Å, and ascorbate, 1.902 Å. Meanwhile a molecule of O_2_ is released, as seen in Figure 15. After elimination of O_2_ and H_2_O_2_ the remaining semiquinone zingerone molecule reacts with an additional ascorbic acid reforming zingerone (Figure 17). The alternative mechanism (green color) is shown in (**e**,**f**). (**e**) species shown in (**b**) interacts with a proton, resulting in H_2_O_2_ formation, well separated by a H-bond from the semiquinone zingerone derivative, 1.819 Å (Figure 12). (**f**) π-π interaction of a second superoxide establishes ring/superoxide interaction, centroid–centroid distance of 2.826 Å, as shown in Figure 16. The resulting semiquinone reacts with a second proton, yielding regenerated zingerone.

The mechanism of zingerone scavenging of superoxide is summarized in Figure 18. Examining the free energy involved at different stages of the mechanism, we see the first proton addition reaction (**b**) **→** (**e**) with ΔG = −106.9 kcal/mol, while the equivalent reaction but using ascorbic acid as the proton source (**b**) **→** (**c**) has ΔG = −61.5 kcal/mol. The final stage of the zingerone reformation process utilizes the second proton (**f**) **→** (**a**) to reform zingerone with (ΔG = −24.1 kcal/mol), whereas the equivalent reaction but using ascorbic acid (instead of proton) (**d**) **→** (**a**) has ΔG = −67.0 kcal/mol. The reaction from (**c**) **→** (**d**) is described in Video S1 in Supplementary Materials.

The dismutation mechanism by which SOD enzymes all reduce O_2_^•−^ to H_2_O_2_ occurs at their active site through the action of redox-active metal ions [26]. In our situation, the molecular structure of the zingerone molecule, through its aromatic ring system coupled with its hydroxyl group, is responsible for the scavenging of two superoxide molecules and the reformation of the zingerone molecule.

## 3. Materials and Methods

### 3.1. Reagents

Zingerone (Indofine Chemical Co., Hillsborough, NJ, USA), ginger powder (SWAD Foods, Artesia, CA, USA), ascorbic acid (Sigma-Aldrich, Inc., St. Louis, MO, USA) were obtained. For electrochemical studies, tetrabutylammonium bromide (TBAB) (TCI Chemicals, Portland, OR, USA) and 99.9% anhydrous Dimethyl Sulfoxide (DMSO; Sigma-Aldrich, Inc., St. Louis, MO, USA) were used.

### 3.2. Method and Equipment

#### 3.2.1. X-Ray Diffraction

A clear colorless block-like crystal of zingerone, C_11_H_14_O_3_, with approximate dimensions of 0.10 mm × 0.16 mm × 0.18 mm, was used for the X-ray crystallographic analysis. A total of 4080 frames were collected on an APEX2 DUO platform X-ray diffractometer from Bruker Advanced X-ray Solutions (Madison, WI, USA) using CuKα radiation, λ = 1.54178 Å. Temperature was maintained at 125 K using a cold liquid nitrogen stream from Oxford Cryosystems (Long Hanborough, Oxford, UK). The total exposure time was 11.33 h. The frames were integrated with the Bruker SAINT 2012 software package using a narrow-frame algorithm. The integration of the data using an orthorhombic unit cell yielded a total of 9280 reflections to a maximum θ angle of 71.87° (0.81 Å resolution), of which 1938 were independent (average redundancy 4.788, completeness = 99.5%, R_int_ = 4.79%, R_sig_ = 3.58%) and 1785 (92.11%) were greater than 2σ(F^2^). The space group was orthorhombic *Pna2_1_* (33) with final cell constants of *a* = 12.3649(4) Å, *b* = 11.6886(3) Å, *c* = 7.1431(2) Å, and V = 1032.38(5) Å^3^, which are based upon the refinement of the xyz-centroids of 4464 reflections above 20 σ(I) with 10.41° < 2θ < 143.7°. Data were corrected for absorption effects using the Multi-Scan method (SADABS). The ratio of minimum to maximum apparent transmission was 0.782. The calculated minimum and maximum transmission coefficients (based on crystal size) were 0.8780 and 0.9300.

The structure was solved and refined using the Bruker SHELXTL Software Package version 2012 [42]. Additional refinement was performed with Olex2 [43,44]. There is one zingerone molecule (formula unit, C_11_H_14_O_3_) in the asymmetric unit, with Z = 4. All hydrogen atoms were found experimentally using information from a difference Fourier map after finding the heavy atoms. The final anisotropic full-matrix least-squares refinement on F^2^ with 183 variables converged at R1 = 3.14%, for the observed data and wR2 = 7.82% for all data. The goodness-of-fit was 1.078. The largest peak in the final difference electron density synthesis was 0.14 e^−^/Å^3^ and the largest hole was −0.150 e^−^/Å^3^, with an RMS deviation of 0.033 e^−^/Å^3^. On the basis of the final model, the calculated density was 1.250 g/cm^3^ and F(000), 416 e^−^. The crystal structure was deposited with the Cambridge Crystallographic Data Centre (CCDC), CCDC2489413 [45].

#### 3.2.2. Electrochemical Studies

A Pine Research WaveDriver 20 bipotentiostat (Pine Research, Durham, NC, USA) with the Modulated Speed Electrode Rotator was used to perform the hydrodynamic voltammetry experiment using a rotating ring-disk electrode (RRDE). The working electrode is the AFE6R2 gold disk and gold ring rotator tip (Pine Research, Durham, NC, USA) combined with a coiled platinum wire counter electrode and a reference electrode consisting of an AgCl-coated silver wire immersed in 0.1 M tetrabutylammonium bromide (TBAB) in dry DMSO in a fritted glass tube. The electrodes were placed in a five-neck electrochemical cell together with means for bubbling the solution with gas. Antioxidant aliquots were introduced in one of these “necks”. Antioxidant aliquots were delivered using an automatic pipette set to deliver the specified concentration. Voltammograms were collected using Aftermath software version 2.1.13189 provided by Pine Research [46]. Thorough cleaning of the RRDE electrode was performed by polishing it with 0.05 µm alumina-particle suspension (Allied High Tech Products, Inc., Rancho Dominguez, CA, USA) on a moistened polishing microcloth to eliminate potential film formation [47].

##### Hydrodynamic Voltammetry (RRDE)

A stock 0.03 M solution of zingerone in anhydrous DMSO (99.9% purity) was used in trials, whereas 0.207 g of ginger powder was added to 10 mL anhydrous DMSO, centrifuged and the supernatant was introduced in the electrochemical cell. The concentration of vitamin C (ascorbic acid) was 0.03 M.

For all the experiments, a solution of previously dried 0.1 M TBAB (1.61 g TBAB) electrolyte completely dissolved in 50 mL anhydrous DMSO was bubbled for 5 min with a dry O_2_/N_2_ (35%/65%) gas mixture to establish the dissolved oxygen level in the electrochemical cell of 50 m. The Au/Au disk electrode was then rotated at 1000 rpm and potential sweep applied to the disk from 0.2 V to −1.2 V and then back to 0.2 V, while the ring was held constant at 0.0 V; the disk voltage sweep rate was set to 25 mV/s.

In an RRDE voltammetry experiment, the generation of the superoxide radicals occurs at the disk electrode, while the oxidation of the residual superoxide radicals (that have not been scavenged by the antioxidant) occurs at the ring electrode.

Reaction 2: Reduction of molecular oxygen at the disk electrode, observed around −0.6 V

Disk Reaction        O_2_ + e^−^ → O_2_•^−^ (2)

Reaction 3 (reverse 2): Oxidation of superoxide radicals at the ring electrode

Ring Reaction        O_2_•^−^ → O_2_ + e^−^ (3)

An initial blank, in the absence of an antioxidant, was run on this solution and the ratio of the ring/disk current was calculated as the “efficiency”. This blank efficiency for zingerone was found to be about 17.5%. Next, an antioxidant aliquot was added using an automatic pipette, the solution bubbled with the gas mixture for 5 min, the voltammogram was rerecorded and efficiency obtained.

In this way, the rate at which increasing concentrations of antioxidant scavenge the generated superoxide radicals during the electrochemical reaction is determined as each additional antioxidant aliquot is added. The results from each run were collected on Aftermath software version 2.1.13189 and represented as voltammograms showing current vs. potential graphs that were later analyzed using MATLAB R2025a [48]. The aliquots used are indicated in related RRDE graphs and are 20, 40, 80, 160, and 320 μL zingerone followed by the addition of 20 and 30 μL of 0.03 M vitamin C to the same electrolytic cell.

Ultimately, the slope of the overall decrease in efficiency with the addition of antioxidant serves as a quantitative measure of the antioxidant activity of each compound. Any decrease in the collection efficiency is expected to be due to the amount of superoxide removed by the antioxidant. The steeper the slope, the greater the rate of change in antioxidant activity and the more effective the antioxidant is at scavenging the superoxide radical.

#### 3.2.3. Computational Study

Calculations and drawings were performed using software programs from Dassault Systemes, Biovia (San Diego, CA, USA). DMol^3^ Density Functional Theory (DFT) was used to calculate energy, geometry and frequencies implemented in Materials Studio 7.0 (PC platform) [49]. We employed the double numerical polarized (DNP) basis set that included all the occupied atomic orbitals plus a second set of valence atomic orbitals, and polarized d-valence orbitals [50]; the correlation generalized gradient approximation (GGA) was applied, including Becke exchange [51] plus BLYP-D correlation. The calculations included Grimme’s correction when van der Waals interactions were involved [52]. For numerical integration of the Hamiltonian matrix elements, all electrons were treated explicitly and the real space cutoff of 5 Å was imposed. The self-consistent field convergence criterion was set to the root mean square change in the electronic density to be less than 10^−6^ electron/Å^3^. During geometry optimization, the applied convergence criteria were 2.72 × 10^−4^ eV for energy and 0.054 eV/Å for force. For correlation with the experimental features obtained through hydrodynamic voltammetry results, we included the effect of DMSO solvent using the continuous model of Dmol^3^ in the calculations [53].

## 4. Conclusions

Since oxidative stress caused by excessive ROS concentration is closely linked to inflammation and subsequent pathological disease states [54], the determination that zingerone in ginger is a very effective scavenger of the superoxide free radical was an interesting outcome. Our results demonstrate how zingerone acts as a strong antioxidant agent through its C4 hydroxyl group. The crystal structure reveals that the hydroxyl group is the fulcrum for strong intermolecular interactions. The cyclic voltammetry experiments show that zingerone, unlike powdered ginger, is a potent antioxidant, capable of scavenging the superoxide anion. Through DFT calculations, we have obtained a mechanism, correlating with experimental data, by which zingerone acts as a strong antioxidant, capable of being regenerated, with the assistance of acidic protons from vitamin C. The chemical reaction is reminiscent of that obtained through the dismutation of superoxide by SOD enzymes into hydrogen peroxide and molecular oxygen. Identification of biologically active phytochemicals which can relieve oxidative stress through their scavenging of excess ROS such as superoxide is a continuing objective of our research group. The results of this work show that zingerone, although just one component in ginger, is an excellent antioxidant and that it can react together with vitamin C, supporting the use of ginger as an effective medicinal agent in traditional medicine.

## Figures and Tables

**Table 1:** Hydrogen bond and close contact distances (Å) and angles (°) for zingerone.

Donor–H		Acceptor–H	Donor–Acceptor	Angle
C5-H5...O1	1.01(3)	2.57(3)	3.489(3)	151.6(19)
O1-H2...O3	0.80(3)	1.94(3)	2.739(2)	175.0(3)
C9-H90...O1	0.96(2)	2.48(2)	3.380(3)	155.2(19)

## Data Availability

Crystal data on zingerone, CCDC2489413, have been deposited at the Cambridge Crystallographic Data Centre (CCDC) and are available at https://www.ccdc.cam.ac.uk/structures. (Accessed on 15 September 2025).

## Supplementary Materials

The following supporting information can be downloaded at https://www.mdpi.com/article/10.3390/ijms262110645/s1.
