# Supplementary material for: Ginger (Zingiber officinale) and Zingerone Antioxidant Properties Studied Using Hydrodynamic Voltammetry, Zingerone Crystal Structure and Density Functional Theory (DFT)—Results Support Zingerone Experimental Catalytic Behavior Similar to Superoxide Dismutases (SODs)
Source: Int J Mol Sci. 2025 Oct 31;26(21):10645. doi: 10.3390/ijms262110645 (PMC12609149; doi:10.3390/ijms262110645)
Supplement: Supplementary file 1 [file ijms-26-10645-s001.zip › supplementary figures.pdf]

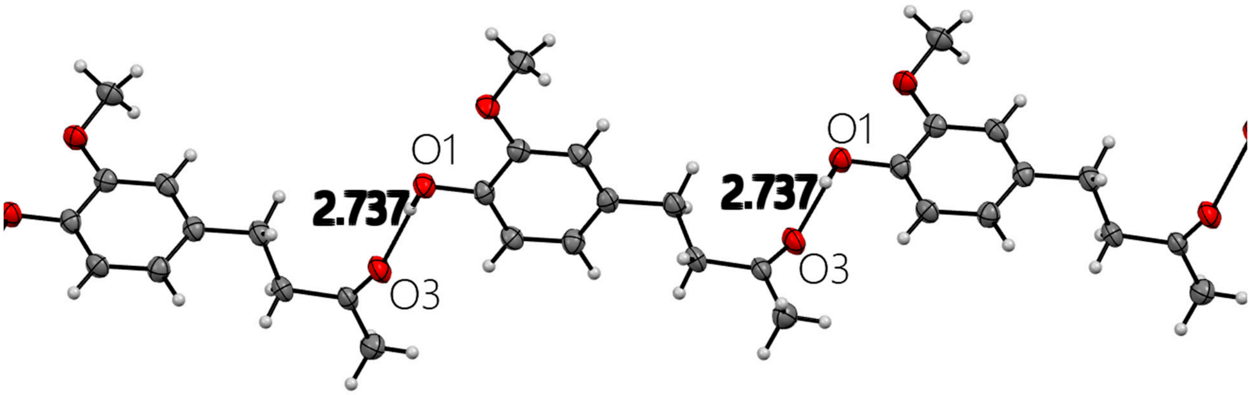

Figure S1. Strong O1-H2...O3 hydrogen bond chain in crystal with distance reported.

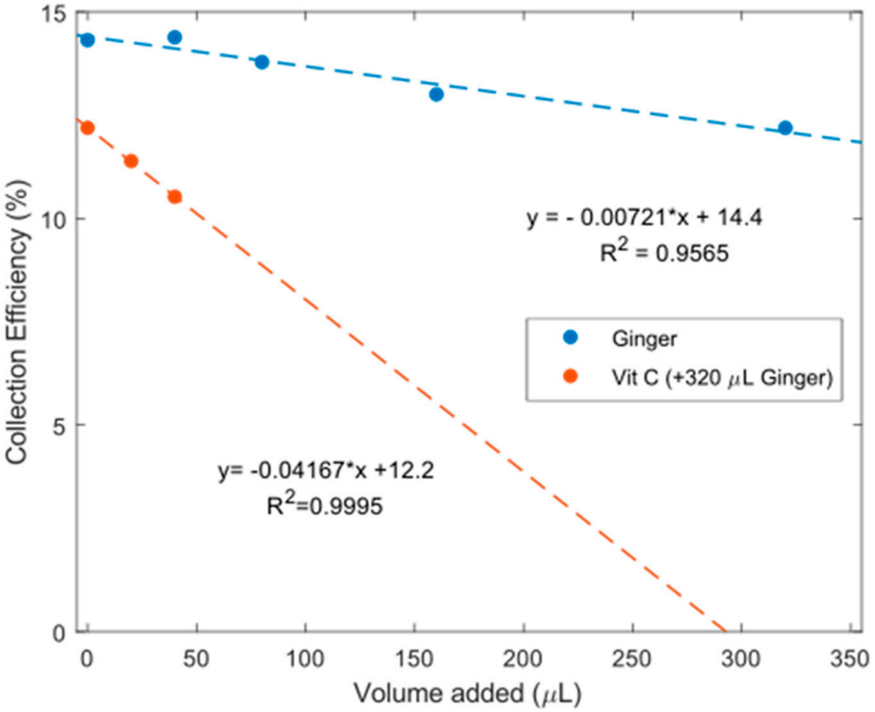

Figure S2. Collection efficiencies for after volume additions of 220 mg/mL ginger and 0.03 M Vitamin C.
